# American Academy of Dental Science

**Published:** 1893-11

**Authors:** 

**Affiliations:** American Academy of Dental Science


### Reports of Society Meetings.

### AMERICAN ACADEMY OF DENTAL SCIENCE.

THE regular monthly meeting of the American Academy of Dental Science was held in the Boston Medical Library Association Rooms, June 7, 1893, at 7.30 P.M., President Brackett in the chair. The subject for discussion was "Orthondontia: Its present status. What are the simplest and most universally applied forms of apparatus, and most efficient retaining fixtures?"

*President Brackett.*—Dr. Smith has kindly consented to open the discussion.

*Dr. Smith.*—I beg you not to get alarmed at this display of charts and models; they are here to help illustrate some points that I could not easily present without their aid.

In order to properly discuss the question propounded by our executive committee, it seems to me necessary to divide it into parts,—

1. Orthodontia: its present status.
2. What are the simplest and most universally applied forms of apparatus?
3. The most efficient retaining devices.

Let us first look a little into the history of orthodontia. In

1541, Egenolff published a book on "Medicine for the Teeth; how to keep them Good and Sound;" and in this book he mentions that form of irregularity produced by the growing of the permanent beside the temporary teeth. And he advises the extraction of the temporary tooth and instructs the patient to press with the finger the permanent tooth, and force it into place.

This, as far as can be ascertained, is the first mention of irregularities. Fox, in 1814, and Calalan, in 1826, wrote extensively on dentistry, but little on regulating devices; and from that time until fifteen or twenty years ago slow progress was made in this important part of our specialty. In 1879, Dr. Kingsley published his work on "Oral Deformities," a book of five hundred and twenty pages, some two hundred pages of which are devoted to the irregularities of the teeth. This was followed in 1888 by the publication of the first volume, to be followed by two more, of a "Treatise on the Irregularities of the Teeth and their Correction," by Dr. J. N. Farrar. This remarkable and thorough work established forever the importance of orthodontia, and tended to make of it a specialty of dentistry.

Dr. Guilford, in 1889, by request of the National Association of Dental Faculties, published his book on Orthodontia, and in this present year has republished it with many valuable additions. This book was published as a text book for use in American dental colleges, and the fact that the author found it necessary to enlarge it, and republish it in so short a time, testifies to the rapid progress being made in the department.

In 1890, Dr. Eugene S. Talbot published his book on the irregularities of the teeth, and goes more deeply into the etiology of the question than any of his predecessors. This book is a valuable acquisition to the literature of orthodontia, and we are encouraged to expect more in the same direction from this able writer. These authors, together with Magill, Angell, Patrick, Bymer, Jackson, Shaw, and others, have given freely of their inventive genius to the construction and improvement of appliances.

Let us now consider what are the simplest and most universally applied forms of apparatus.

In answer to this part of the question, I can only say what *I* consider to be the simplest and with me the most universally applied forms of apparatus. And to better present my point I will refer to these models and charts. But before doing so I wish to call your attention to the fact that in bringing these models and these charts here to-night, I do not intend showing completed cases of irregularities; that is, I do not show the models before regulating, the appliance used, and the condition after correction. I have brought parts of different cases in order to show the appliance for producing a certain movement. And first, as to moving instanding teeth.

This illustration shows an upper lateral incisor striking inside the lower incisor teeth. There is an opinion held by many that in order to bring that tooth back into place it is necessary to break the occlusion; in other words, the appliance must be so constructed that no tooth can strike in front of the tooth to be moved. I never pay any attention to the occlusion in moving a tooth. If it were true that the teeth were constantly locked together, the occlusion would have to be considered. But the fact is that we never have our teeth together except when eating, and this fact shows how unimportant this consideration is. In the case before us, therefore, we pay no attention to the articulation of the teeth, but simply adjust to the lateral incisor the necessary appliance. This case was completed in seven days, without pain or inflammation. The appliance used in such cases generally consists of what I term a Magill band. This is a ferrule similar to the band which is used with the Richmond crown, fitted to the tooth and cemented to place. To this Magill band, on the labial surface, is attached a post with a screw-thread cut around it. This post passes through a bar, which rests upon, in this case, a central incisor and a cuspid.

By the side of this post is what I term a leader. It is simply a straight piece of gold, going from the Magill band through the bar, by the side of the post, to prevent the bar from twisting as you turn the nut. On the end of the screw-post a nut is placed, and when this nut is turned up the teeth move apart and the incisor is brought forward to place. A wrench is given to the patient and the nut turned a little every day. With this appliance the pain is absolutely in the hands of the patient. It is not necessary to go into a physiological explanation of the change that takes place. I simply mention it as one method, and a positive one, of moving instanding teeth into line.

In this second case, as the lateral was very much crowded, instead of making a ferrule, a gold plate was made to fit the labial surface of the tooth, and secured to it by a ligature wound around the tooth, commencing at its neck and carried to the cutting-edge. That case was also completed in seven days without any trouble; and when the tooth was in place, there was no need of a retaining fixture. This chart shows a lateral incisor which was twisted; it was turned a little as it was brought to place. The appliance used contained two posts and two screws. In other respects it was the same as the one first described.

Another model that I will show you is that of a second bicuspid placed inside the arch, the first bicuspid and molar being in contact. To look at that model and see the appliance, we would hardly believe that it brought the bicuspid into place without any extraction, but, nevertheless, it did. This was a case that was under the immediate control of Dr. Banfield, who used the appliance and can vouch for its results.

When outstanding teeth are to be moved to place, the occlusion need not be considered. A very simple arrangement for this purpose is the Jackson appliance, which is made of piano-wire. The wire impinges upon the tooth and forces it to place. Years ago piano-wire was mentioned by Dr. Coffin, whose name is associated with the Coffin plate. Dr. Jackson sometimes constructs the whole appliance of piano-wire, or uses it in connection with a rubber plate.

For spreading arches I will give you some of the simpler appliances. The easiest and simplest appliance is the Coffin plate. When once applied, it is sawed through the centre and the wire pulled apart, so that the spring acts upon the vulcanite and spreads the arch. The difficulty I have had with the Coffin plate is that in short teeth it is almost impossible to make the plate stay up, and in such cases I use the Shaw plate. In nearly all contracted arches you most need to spread the teeth in the anterior part of the mouth. It is very rare, indeed, to find that the arch needs spreading at the molars. The Shaw plate spreads the arch anterior to the molars more than at the molars. Here is an illustration of it. This is a vulcanite plate; here are the wings which are carried apart, and here are the jack-screws supplying a positive force. Here is a combination Shaw plate for a case in which you do not care to move the molars, or at least but very little. In this is made what I term a "movable resistance:" the power is applied on the outstanding tooth. There are many cases where the moving back of projecting teeth involves an important question of resistance. The making of a resistance of any one of the back teeth is always attended with some risks, on account of the disposition of posterior teeth to move forward. As a result, when you have finished regulating, you may find an impaired occlusion. In my experience with such cases I have found that, after my regulating was completed and the æsthetic part was satisfactory, I had tipped a molar up or down or driven a tooth into the socket, so that the articulation was injured.

In this case, the model of which I now show you, I made a resistance of a skull-cap. I first fitted Magill bands to the molars and central incisors. On the buccal surfaces of the molars were attached two lugs, through which was a hole for the passage of a gold wire, which extended in front of the incisor bands and was kept from slipping by four projections attached to these bands. A bib or bar extended across the outside of the mouth and rested on the wire before mentioned. To the bib were attached four strong elastics, which extended to and found attachment on the skull-cap already described. This was worn during the night only, and the movement gained was held during the day by an elastic, extending from a slight enlargement on the wire to the lugs on the molars. The projecting incisors were carried back in a comparatively short time, and this was accomplished without pain and with but little discomfort.

Another process of moving teeth which is much in favor with me is this. I make a vulcanite plate covering the palatine arch, to which is attached piano-wire, which fits all the teeth excepting the one I wish to move. In this way the teeth are all tied together and make a resistance that is immovable. I then wedge the tooth which I wish to move, to the desired position.

Now, for torsion of teeth, this chart represents a medium case. Here I used piano-wire in connection with a band, as here illustrated. The spring of the piano-wire turns the tooth into place. In using piano-wire, care must be taken not to have it too large.

Where teeth do not erupt as readily as they should, there are many ways of assisting the process. The simplest, as shown in this illustration, is an appliance made to impinge upon the tooth which you wish to elongate.

Where there is a projection of the lower jaw, the only method to bring it back to place is to use the skull-cap with powerful elastics. There are different theories as to how the jaw is carried back. Some hold that the ramus is bent. But I think it is accomplished by the carrying back of the condyle into the glenoid cavity.

If you have moved an outstanding tooth to line and a retaining fixture is necessary, here is an appliance that can be used. A lateral incisor has been carried to place and is retained simply by a Magill band with two prongs attached to it, one on the inside and the other on the outside. Another retaining fixture which was worn for seven months was made by striking up caps to fit over several teeth and cementing them in place. The gold was somewhat unsightly, and it was afterwards made less cumbersome with the use of a little wire crib. In case you have spread an arch and want to hold it in place, it can be done by putting in a vulcanite plate covering the arch.

We sometimes have a deformity of the teeth caused by a deformity of the alveolus. In the case which I show you the jaw is farther down on one side than it is on the other; and I learned from the mother that the child's head was very much compressed and broken by the forceps at birth.

When one comes to study the many different appliances which are set before him for the regulation and retention of teeth, he is led to conclude with Hamilton that a man's genius is shown not so much in inventing new devices, as in choosing from the devices already invented.

*President Brackett.*—Gentlemen, you have all listened with much interest and profit to this discourse from one who knows practically of what he speaks. I have no doubt that Dr. Smith will be ready to make plainer anything that is not fully comprehended, and the whole subject as stated upon the card is before you for discussion.

*Dr. Williams.*—Dr. Smith in his discourse referred to what Dr. Jackson called the "crib." I may be able to throw a little light on the history of it. About forty years ago I saw a case of that sort on a patient who had been in Paris and who brought it over with her. It was put on by Dr. Evans, and that gave me the idea of what I called the "basket" framework as a basis for various kinds of regulating, and I have used it extensively ever since. When Dr. Jackson called my attention to the idea and supposed he invented it,—and he probably did invent it, for it sometimes happens that a person will think of something that he does not know already exists,—I wrote to him reminding him of these things, so that he might know how far back the idea could be dated. A specimen of it was made by Dr. Merrill when he graduated at the Harvard Dental School in 1871, and placed in the museum, and is probably there now.

*Dr. Smith.*—Dr. Payne reminds me that I have given the impression that the Shaw plate for spreading must necessarily cover the teeth. It can be adjusted merely on the lingual side when necessary for the comfort of the patient. I make a great point of not hurting patients in regulating their teeth.

*Dr. Williams.*—I used the ferrule that you call the "Magill band" when I was quite a youngster with Dr. Keep. That was before the phosphates were invented, and I arranged it so as to tie it on and thus keep it in place.

*Dr. Clapp.*—I cannot let the opportunity pass without expressing my very high appreciation of the talk that we have had to-night. The subject has been put before us in such a clear and comprehensive manner that we must be very dull indeed if we can go home without receiving much benefit from it. So far as I have used the appliances spoken of, I can testify to their efficacy.

*Dr. Banfield.*—It may not be generally understood that in the adjustment of some of the plates described, the teeth need separating. Will Dr. Smith please explain?

*Dr. Smith.*—It is necessary to put in one ligature or wedge, so as to let this thin band of No. 30 gold pass. It requires but little separation, and I prefer to separate rather than file the teeth.

*Dr. Batcheller.*—Dr. Smith spoke of wedging teeth forward. May I ask what he uses for large wedges?

*Dr. Smith.*—In some cases I use a piece of cottonwood, in others hickory. These wedges are fitted nicely, and the pain is entirely under your control, just as it is with a screw. In moving a tooth it is our object to have that movement take place with the minimum amount of pain and inflammation. As long as the change is physiological it is a healthy one, but when it becomes pathological you run the risk of a dead tooth. To-day with the modern appliances the teeth can be regulated with but little pain or discomfort to the patient.

*Dr. Cook.*—I am very much indebted to Dr. Smith for presenting this subject in the thorough manner in which he has. I could not help thinking, while he was speaking, that if I had received such plain instruction when at the dental school I would not have had so much difficulty in understanding this part of practice.

I use the rubber pin inserted in a conical hole for moving out a tooth. I drill the hole right through the plate with a square-pointed fissure-drill. The rubber is prepared by taking two thicknesses of rubber and putting them into a vulcanizer between two pieces of German silver. I file this vulcanite into proper shape, and insert it in the plate so that it will impinge the neck of the tooth and force it out.

For drawing the front teeth back I use a jacket-plate. A hole is made in it by vulcanizing a piece of piano-wire in its substance, then drawing the piano-wire out. A gold wire is inserted in its place and a rubber elastic attached to the gold wire; this elastic serves as the force for pulling the tooth into line.

*Dr. Fillebrown.*—Referring to the matter of history, I might speak of what Dr. Smith calls the "Magill band," which, by the way, is a good name for it. The first man I ever heard mention it was Dr. Shepard, who showed it before the New England Dental Society, about 1865, long before oxyphosphate was known.

I used a Farrar appliance in a case which was recalled by Dr. Smith's speaking of the disposition of posterior teeth to move forward. An inferior cuspid was quite out of line, and I wanted to move it back. To do this I removed the first bicuspid, and made a Farrar band that enveloped the two molars and the remaining bicuspid. The cuspid was clasped with a band regulated by a screw. Those three teeth were to be used as a resistance, and I gave my patient a wrench, with the necessary instructions. But, instead of the cuspid starting a bit, the three posterior teeth came forward into the space left by the bicuspid.

I use the screw oftentimes for moving instanding teeth outward. I don't know who invented it, but I have used it as far back as 1883. A jacket-plate is made and a small screw inserted behind the tooth to be moved. This is turned up about half a turn each day. I have moved a lateral incisor out with this appliance in about a week.

I have lately used the rubber pin set in a tapering hole in a rubber plate. It slips up against the tooth and moves it out very nicely.

I have always looked favorably upon piano-wire for moving the teeth in, though I have relied principally on the jacket-plate. In using a jacket-plate one must be sure and have a perfect resistance; if you do not, the plate will be thrown out.

I was much pleased to learn the fact, which Dr. Smith has mentioned, that you can move an instanding tooth without making an opening for it. My feeling was that it would impinge so much as to make it impossible to get it into place; but I am happy to know that it does not, and will be pleased to try his method.

*President Brackett.*—Unless there are further remarks, it would seem appropriate to proceed to the next subject of discussion that has been put before us. The subject is, "What materials are best for temporary fillings to be retained for a minimum of three years?"

*Dr. Stevens.*—I would suggest that most of the permanent fillings that are put in do not last as long as that.

*Dr. Fillebrown.*—I should call a filling permanent if it lasted three years.

*Dr. Williams.*—I once put in some temporary fillings for obtunding purposes, intended to last for three months, and they remained twenty-eight years. I then took them out and filled with gold. The cavities were between the lower incisors; they were completely protected from wear, and no subsequent caries occurred.

*Dr. Banfield.*—I have recently seen Weston's cement fillings that I inserted in crowns of molars eight years ago. They are now in as good condition as when inserted.

*Dr. Eames.*—I think it would be well to have a general understanding as to what we mean by temporary fillings. Years ago I supposed that temporary fillings were associated with certain materials, such as gutta-percha, cement, etc.; but I now understand that a gold filling may remain in a cavity a much shorter time than gutta-percha. For instance, some time ago I saw a well-preserved red gutta-percha filling which had been in the mouth for seventeen years. It was on the lingual surface of a superior central and lay well up under the gum. The filling was flush with the edges of the cavity, and needed no repair. It seems to me that a temporary filling is one which is inserted for a limited time, with the intention that some other filling will take its place in the future.

*Dr. Williams.*—One great trouble I have found in using temporary fillings is that they are likely to be forgotten by the patient, and are supposed to be permanent, while the original intention was to prepare the cavity for a permanent filling. I often call such fillings a dressing, and tell patients "I am putting this on as I would a bandage on a wounded finger. It is not intended to remain; you must come and have a more durable put in." I do not call this a filling, but a treatment.

*President Brackett.*—We come to "Incidents of Practice and Presentation of Specimens."

*Dr. Allen.*—I have a couple of models here which may interest the members,—two cases of hyperostosis, one of the lower jaw, and the other of the hard palate. The teeth in each case are in a perfectly healthy condition.

*President Brackett.*—Probably there is little tendency of the teeth to decay.

*Dr. Allen.*—Very little. The lower case has been progressing for twenty-five years, and there has been a slight increase in the size of the tumor during the last five years. The size of the tumor in the other case has not changed perceptibly during the past six years, in which I have had frequent opportunities for observing it.

*Dr. Williams.*—I saw a case of that sort on the upper right jaw which grew so large that it impeded the gentleman's speech. It was cut out and never returned. I also had another case on the lower right jaw, and in this case the protuberance was a small one. It had existed for three or four years, and occasionally there was inflammation. I made an incision and scraped off a little piece of exfoliated bone, and then it healed up.

*Dr. Clapp.*—Some two or three years ago one of my patients came into the office considerably excited, and said that her husband was very much depressed and alarmed on account of having discovered a hard tumor in his mouth. She said that his physician had seen it, and rather increased the alarm. I examined the case and found a growth not more than one-half or two-thirds as large as the one Dr. Allen has shown, and I told the gentleman that he had probably carried it there for twenty years and had just discovered it. He now takes no notice of it, and it has not increased in size since that time.

There is a case which I wish to speak about which is fresh in my mind, as the patient was in my chair to-day. It is a boy thirteen or fourteen years of age, who is very healthy, and has been in at least average health all his life; but his molars, bicuspids, and incisors are simply riddled with cavities. You have all seen the same character of teeth; if there is the least defect in the enamel, on following that up you will find a large cavity beyond. This condition is in contrast with that found in the boy's brother, who is a year or two older and who has for years been very much out of health. His system has been upset some way or other, I don't know how, but his nutritive functions are all out of order, and he isn't able to go to school except a part of the time. He came to my office the other day and I examined his teeth and found them in very good condition, and during the past six or eight years I have put in on an average not more than one or two fillings a year, while his brother has teeth which look as though they had a charge of fine shot put into them.

*Dr. Banfield.*—I have a patient, a boy of sixteen years, whose teeth are riddled with cavities in the manner of which Dr. Clapp speaks. He has an older brother whose health is not as good, but whose teeth are in a much better condition, perhaps up to the average. I would like to ask Dr. Clapp if in his case he advised filling these teeth with gutta-percha or cement, or if he would discourage the boy in trying to save his teeth?

*Dr. Clapp.*—I have used nothing but cement fillings. They last very well, but an enormous number of new points of decay seem to develop at every examination. Of course I occasionally have to refill the cavities that I have filled with cement. I certainly try to encourage the boy and not discourage him.

*Dr. Smith.*—Have you tested the secretions in the mouth?

*Dr. Clapp.*—No, I have not. It seems to me that the cause was the improper nutrition of the teeth during their formation. The decay is very marked, as though the fissures were not properly calcified when the teeth were growing.

*President Brackett.*—The chair would ask Dr. Clapp if he has knowledge of the dietary habits of the child,—whether it is suitably fed at suitable intervals, and is not kept constantly supplied with confectionery by an injudicious relative.

*Dr. Clapp.*—I have not made any inquiries. The family consists of four children, and the mother is very careful and a woman of good common sense; but so far as my experience goes in trying to get at the dietary habits of children, it is simply a farce. They will tell you almost anything.

*Dr. Banfield.*—Dr. Clapp says that he has used cement. I would like to ask if he thinks that wears as well as gutta-percha?

*Dr. Clapp.*—I have no fault to find with it in this case.

*Dr. Banfield.*—With the patient that I have I first tried cement, but as it dissolved badly at the cervical walls, I have put in gutta-percha for the last few years, using in most places Owens's. I find that it does better in his mouth than cement. Even gutta-percha after awhile disintegrates and needs replacing. There is not a sound tooth in the boy's mouth, and if it were not that he was faithful he would long ago have had a number of exposed pulps.

*Dr. Allen.*—Although it is out of the regular order, I move that we now listen to Dr. Fillebrown, who has a new method of administering ether.

*Dr. Fillebrown.*—The chief difficulty which we have had in etherizing patients is that we have frequently been obliged to stop work in order to re-etherize, and it takes longer to etherize than it does to do an ordinary operation. Last fall I became acquainted with Dr. Packard's method of etherizing, which consists in supplying an atmosphere with a definite amount of ether vapor in it. It occurred to me to extend this principle and make the apparatus on a large scale. I used a wash bottle, such as is made for purifying gas. You will see that there are two tubes passing through the cork of the bottle, which contains ether. Into the long tube I pass a column of air driven by the Fletcher bellows, and this stop-cock controls the amount of air which shall pass through the ether.

The vapor issues from the shorter tube, which can be of any good length, and the whole is placed in such a position that it will be near the face of the patient and still far enough off so that it will not excite inflammation. Both hands are by this method free for work. Anæsthesia can be maintained as long as you please, so that you can excise a nerve or perform staphylorrhaphy. I first experimented with the apparatus on a student who liked the fun of being anæsthetized, and I maintained the condition as long as I pleased. Twice since that I have done quite extended surgical operations in the mouth, and maintained the anæsthesia successfully. In the latter cases I induced anæsthesia by the older methods, and then maintained it with this method. In one case I found the anæsthesia so deep that I was obliged to reduce the strength of the vapor to prevent too profound effects. I believe this to be a great advance in the matter of anæsthetizing patients for operations in the mouth. The objection might be raised that the operator himself would be affected by the ether which permeates the air. I can only say I have experienced no difficulty in that direction.

William H. Potter, D.M.D.,
*Editor American Academy of Dental Science.*

